# Association of dysfunctional breathing with health-related quality of life: A cross-sectional study in a young population

**DOI:** 10.1371/journal.pone.0205634

**Published:** 2018-10-11

**Authors:** Ji-Myung Ok, Young-Bae Park, Young-Jae Park

**Affiliations:** 1 Department of Human Informatics of Korean Medicine, Graduate School, Kyung Hee University, Dongdaemun-gu, Seoul, Republic of Korea; 2 Department of Biofunctional Medicine and Diagnostics, College of Korean Medicine, Kyung Hee University, Dongdaemun-gu, Seoul, Republic of Korea; 3 Department of Diagnosis and Biofunctional Medicine, Kyung Hee University Hospital at Gangdong, Gangdong-Gu, Seoul, Republic of Korea; Public Library of Science, UNITED KINGDOM

## Abstract

Symptomatic hyperventilation (SH) is a pathological condition that manifests with breathlessness, dyspnea, light-headedness, anxiety, and paresthesia. However, little is known about the prevalence of SH and its association with health-related quality of life (HRQoL) in a young population. The Nijmegen questionnaire (NQ), which measures severity of SH, had not previously been cross-culturally translated into Korean. In this study, the NQ was cross-culturally translated into Korean (KNQ), using translation and back-translation methods. To examine the reliability and validity levels of the KNQ, as well as its association with HRQoL, 237 college students (21.38 ± 2.45 years) were asked to complete the KNQ, the Korean version of the general health questionnaire (K-GHQ-30) and the short form-36 (K-SF-36). The KNQ showed satisfactory reliability (Cronbach’s α = 0.878). In the construct validity test, four factors (neuropsychological, respiratory, neurogastrointestinal, and neuromuscular) were extracted (% of total variance = 59.8). Using a KNQ cut-off score of 23 points, the prevalence of SH was 22.8%. Physical and mental HRQoL levels estimated by the K-GHQ-30 score and the 8 subscale scores of the K-SF-36 were lower in the SH group than in those of the non-SH group. It is concluded that the cross-culturally translated KNQ is reliable and valid, and management of SH may prevent a reduction in physical and mental HRQoL in a young population.

## Introduction

Hyperventilation is a condition in which arterial carbon dioxide (CO_2_) is eliminated excessively by respiration beyond the demands of metabolism, resulting in a wide range of symptoms [1]. Hyperventilation is associated with a variety of pathological conditions, including respiratory and cardiovascular diseases and psychiatric disorders [1]. Although a low arterial CO_2_ level is regarded as the primary criterion for hyperventilation [2], some studies have reported hyperventilation cases with normal CO_2_ levels [3]. Therefore, together with arterial CO_2_ levels, the presence of hyperventilation-related symptoms, including breathlessness, dyspnea, light-headedness, anxiety, and paresthesia, are now regarded as hyperventilation parameters.

The Nijmegen Questionnaire (NQ) was developed at Nijmegen University in the 1980s to evaluate symptomatic hyperventilation (SH), and the NQ was reported to possess satisfactory reliability and validity [4]. Since the development of the NQ, the clinical severity of SH estimated by the NQ has been associated with a wide variety of diseases, including asthma, anxiety disorder, and chronic fatigue syndrome [5–9]. SH was also reported to occur in patients with prior diseases as well as in the general population, reflecting the diversity and lability of SH. Thomas et al. used the NQ to determine the prevalence of SH in adults who had previously been treated for asthma but did not have asthma at the time of the study and found a SH prevalence of 9.5% [10]. In terms of the general population, Shimmenti reported a prevalence of 27% among the general female population [11]. Although SH may be especially problematic in the third to fourth decade of life, younger people were also affected by SH. Gridina et al. reported that the prevalence of SH in children and adolescents (aged 1 to 17 years) was 21%, based on results of the Hyperventilation Syndrome Ambroise-Paré Enfant questionnaire [12]. However, few studies have addressed the prevalence of SH in a young population. Given that SH is underdiagnosed by general practitioners, and unknown even by sufferers, it is possible that a sizable portion of young people may be living with unmanaged SH. Some studies have reported that the health-related quality of life (HRQoL) of patients with hyperventilation is impaired [8,13,14]. These findings suggest that unperceived SH may be associated with a reduction in HRQoL in a young population, similar to that found in patients. Therefore, the purpose of our study was to determine the prevalence of SH using the NQ, and to examine the association of SH with HRQoL in a young population.

Since the development of the original NQ, this survey has been translated into many other languages, including Greek, Norwegian, and Persian [3,15,16]. However, it has not been cross-culturally translated into Korean nor validated in this population. Therefore, together with determining the prevalence of SH and its association with HRQoL, we conducted a cross-cultural translation of the NQ into Korean (KNQ) in accordance with a published guideline [17], and examined its reliability and validity.

## Materials and methods

**Fig 1:**
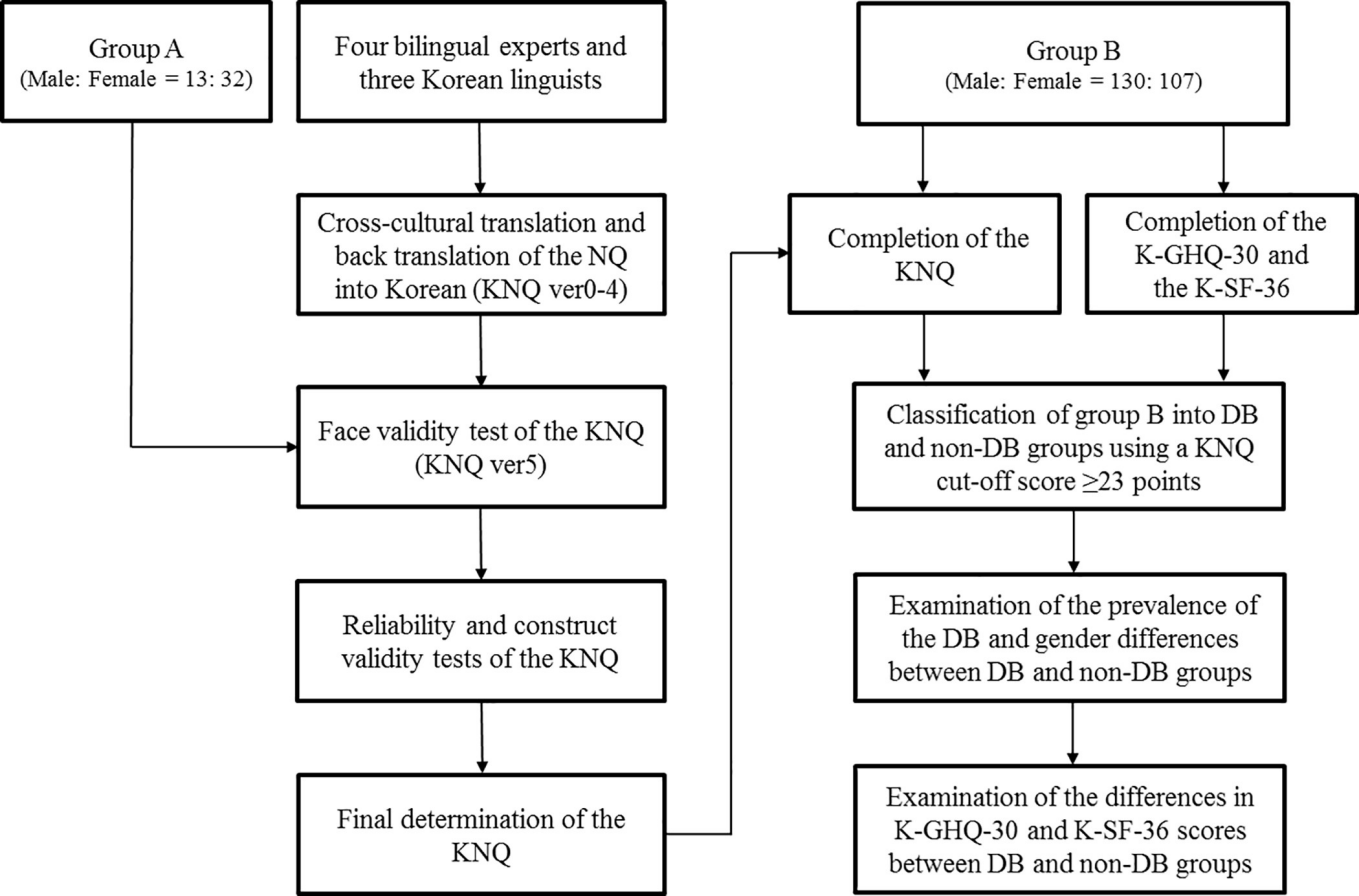
Flowchart of the study design. NQ = Nijmegen Questionnaire, KNQ = Korean version of the Nijmegen Questionnaire, K-GHQ-30 = Korean version of the General Health Questionnaire-30, K-SF-36 = Korean version of the Short Form-36, DB = Dysfunctional Breathing.

A flowchart of the study design is presented in Fig 1. Two groups of volunteers participated in this study, groups A and B. Group A consisted of 45 apparently healthy participants: 13 males and 32 females with an age range of 24 to 55 years. The inclusion criterion for group A was the ability to read and understand the KNQ. Participants in group A were asked to complete the KNQ, and their responses were used to determine the face validity of the instrument. Group B consisted of 237 college students: 130 male (mean age of 21.35 ± 1.87 years) and 107 female students (mean age of 21.41 ± 3.02 years). The inclusion criteria for group B were being a college student and having no impediments to daily life caused by psychological or respiratory problems. Participants in group B were asked to complete the KNQ and the Korean versions of the General Health Questionnaire-30 (GHQ-30) [18], and Short Form-36 (SF-36) [19]. Data collected from group B were used to determine the reliability and construct validity of the KNQ, and to examine the association between SH and HRQoL levels estimated by the GHQ-30 and SF-36. The protocol for this study was approved by the Kyung Hee University Institutional Review Board (KHSIRB-15-010RA). Informed consent was obtained from all participants.

### Scoring of the NQ, GHQ-30, and SF-36

The original NQ consists of 16 questions related to SH. For each question, the respondent is asked to rate the occurrence of the symptom on a 5-point Likert-type scale: 0 = never, 1 = rarely; 2 = sometimes; 3 = often; 4 = very often. A total score of 23 or higher is considered indicative of SH. The NQ has a sensitivity of 91% and a specificity of 95% [4]. The GHQ-30 is a screening tool based on self-reporting of psychiatric symptoms in the general population [18]. The GHQ-30 uses a dichotomous scoring method (0-0-1-1) transformed from four response items (0-1-2-3), with a maximum score of 30 points. Higher scores on the GHQ-30 indicate a lower HRQoL level that is associated with higher psychological distress. The SF-36 is a measurement tool used to assess general level of health [20]. It consists of one question on change in health status and 35 additional questions divided into eight, individually scored subsections: physical functioning (PF), social functioning (SF), role limitation-physical (RP), role limitation-emotional (RE), mental health (MH), vitality (VT), bodily pain (BP), and general health (GH) [19]. Subscales in the SF-36 are scored based on question type: dichotomous (0–100), three point (0-50-100), five point (0-25-50-75-100), or six-point (0-20-40-60-80-100). Unlike the GHQ-30, lower scores in the eight subscales of the SF-36 indicate a lower HRQoL [19]. The Korean versions of the GHQ-30 (K-GHQ-30) and SF-36 (K-SF-36) were previously validated [18,19].

### Cross-cultural translation of the NQ in Korean

The cross-cultural translation performed in this study followed published guidelines and included quality control and back-translation steps, as well as face validity testing [17]. The initial forward translation into Korean was performed independently by four bilingual experts (KNQver0). The KNQver0 was then aggregated into one translation by five Korean medical doctors (KMDs) who took cultural, linguistic, and emotional factors into account (KNQver1). Next, two bilingual translators who did not previously participate in the KNQver0 process conducted a back-translation of KNQver1 into English (KNQver2). After back-translation, the same five KMDs who contributed to KNQver1 reviewed KNQver1 and KNQver2, and prepared KNQver3 with consideration of the discrepancies between KNQver1 and KNQver2. Three Korean linguists reviewed KNQver3 and corrected all typographical and grammatical errors to create KNQver4. Finally, a face validity test was conducted, in which group A was asked to complete KNQver4 and list all semantic and grammatical obscurities that they found while responding. Of the 45 participants, 9 participants indicated ambiguity in the phrase “tight feeling around mouth,” and the same KMDs revised KNQver4 based on results of the face validity test, and established KNQver5. After this, results from KNQver5 testing were used to determine whether the KNQ showed satisfactory reliability and construct validity.

### Statistical analysis

The reliability of the KNQ was examined using Cronbach’s α coefficient. A Cronbach’s α value over 0.6 is considered indicative of acceptable reliability [20]. The construct validity of the KNQ was examined using a principal component analysis (PCA) with varimax rotation. The Kaiser criterion was used, and only factors with eigenvalues greater than 1.0 were retained. According to a previous study [4], we assigned the subjects who scored 23 points or more on the KNQ to the SH group, whereas those who scored less than 23 points were assigned to the non-SH group. Gender differences between the SH and non-SH groups were examined by chi-square (χ^2^) test. Differences in the K-GHQ-30 and K-SF-36 subscale scores between the SH and non-SH groups and between genders were examined by two-way ANOVA. All statistical analyses were conducted using SPSS 18 for Windows (SPSS, Inc. Chicago, IL, USA). Values are presented as the mean ± standard deviation, and *P* < 0.05 was considered significant.

## Results

Table 1 lists descriptive characteristics of the KNQ, K-GHQ-30, and eight K-SF-36 subscale scores. Table 2 presents the prevalence of SH and non-SH by gender. Based on the 23-point cut-off for the NQ [4], 54 college students were categorized in the SH group, indicating a prevalence of 22.8%. There was no difference in SH prevalence based on gender. Table 3 provides the results of the reliability test of the 16 symptoms listed in the KNQ, including their Korean translations. In the final revision of the KNQ, five items (“Dizzy spells”, “Faster or deeper breathing”, “Short of breath”, “Bloated feeling in stomach”, and “Tight feelings round mouth”) were determined to contain words in both native Korean and Sino-Korean, since words from both these languages are regularly used inter-changeably in Korea [21]. The overall Cronbach’s α value was 0.878, indicating that the KNQ demonstrated satisfactory internal consistency [20]. This score was consistent with the reliability values of previous cross-cultural translations into other languages, which ranged from 0.70 to 0.9 [3,15]. Although Cronbach’s α increased after removing “tingling fingers” and “cold hands or feet” (0.879 and 0.884, respectively), the effect was slight (0.001–0.006). Therefore, the following analysis was conducted with all 16 terms included. In the construct validity test by PCA, four factors were extracted, and the percent of total variance was found to be 59.761% (Table 4). The four extracted factors were as follows: neuropsychological (factor 1), respiratory (factor 2), neurogastrointestinal (factor 3), and neuromuscular (factor 4).

**Table 1:** Descriptive characteristics of the KNQ, K-GHQ-30, and subscales of the K-SF-36.

Instrument	Subsection	Minimum	Maximum	Mean ± SD
KNQ (score)		0	43	15.61 ± 9.42
K-GHQ-30 (score)		0	27	8.41 ± 6.21
K-SF-36 (score)	PF	40	100	91.71 ± 11.58
	RP	0	100	78.38 ± 32.53
	BP	0	100	76.74 ± 19.43
	GH	10	100	58.88 ± 18.25
	VT	0	100	49.89 ± 17.30
	SF	0	100	76.00 ± 20.35
	RE	0	100	77.92 ± 36.50
	MH	5	100	65.15 ± 16.66

KNQ = Korean version of the Nijmegen Questionnaire, K-GHQ-30 = Korean version of the General Health Questionnaire-30, K-SF-36 = Korean version of the Short Form-36, PF = Physical functioning, RP = Role-physical, BP = Bodily pain, GH = General health, VT = Vitality, SF = Social functioning, RE = Role-emotional, MH = Mental health.

**Table 2:** Participants with and without symptomatic hyperventilation by gender.

Gender	Group		Total	χ^2^ value	*P* value
	Case of non-SH (%)	Case of SH (%)			
Male	106 (44.7)	24 (10.1)	130 (54.9)	3.059	0.089
Female	77 (32.5)	30 (12.7)	107 (45.1)		
Total	183 (77.2)	54 (22.8)	237 (100.0)		

SH = Symptomatic hyperventilation.

**Table 3:** Results of the KNQ reliability test (participants, n = 237).

English (Korean [Sino-Korean])	Cronbach’s α when each item was removed	Overall Cronbach’s α
Chest pain (가슴부위 통증)	0.871	0.878
Feeling tense (긴장된 느낌)	0.864	
Blurred vision (시야가 흐릿함)	0.875	
Dizzy spells (현기증(어지럼증))	0.870	
Feeling confused (혼란스러워 이해나 판단이 어려운 느낌)	0.867	
Faster or deeper breathing (숨(호흡)이 점점 더 빨라지거나 깊어짐)	0.870	
Short of breath (숨(호흡)이 짧음)	0.868	
Tight feelings in chest (가슴이 조이는 느낌)	0.868	
Bloated feeling in stomach (윗배 더부룩함(팽만감))	0.874	
Tingling fingers (손가락이 따끔거리는 느낌)	0.879	
Unable to breathe deeply (숨을 깊게 못 쉼)	0.869	
Stiff fingers or arms (손가락이나 팔이 뻣뻣함)	0.873	
Tight feelings round mouth (입 주위가 조이는(당기는) 느낌)	0.873	
Cold hands or feet (손이나 발이 차가움)	0.884	
Palpitations (심장이 두근거림)	0.863	
Feelings of anxiety (불안한 느낌)	0.865	

KNQ = Korean version of the Nijmegen Questionnaire.

**Table 4:** Results of the KNQ construct validity test.

Item	Factor 1	Factor 2	Factor 3	Factor 4
Chest pain	**0.771**	-0.001	0.128	0.145
Feelings of anxiety	**0.730**	0.163	0.274	0.162
Feeling tense	**0.725**	0.255	0.289	0.053
Palpitations	**0.672**	0.286	0.235	0.200
Tight feelings in chest	**0.627**	0.382	0.078	0.127
Short of breath	0.132	**0.859**	0.125	0.214
Faster or deeper breathing	0.140	**0.808**	0.203	0.058
Unable to breathe deeply	0.312	**0.674**	0.034	0.266
Feeling confused	0.374	**0.504**	0.396	0.033
Blurred vision	0.161	0.177	**0.666**	0.000
Dizzy spells	0.253	0.245	**0.665**	0.046
Cold hands or feet	0.056	0.025	**0.648**	0.094
Bloated feeling in stomach	0.272	0.015	**0.594**	0.243
Tingling fingers	0.020	0.004	0.119	**0.820**
Tight feelings round mouth	0.249	0.312	0.037	**0.604**
Stiff fingers or arms	0.252	0.246	0.140	**0.589**
% variance	19.155	16.682	13.406	10.517

KNQ = Korean version of the Nijmegen Questionnaire. Bold values indicate the highest factor loading values of the four factors.

Table 5 lists differences in the K-GHQ-30 and K-SF-36 subscale scores between the SH and non-SH groups, and between genders. The mean K-GHQ-30 score in the SH group was higher than that in the non-SH group, and the means of the eight subscale scores of the K-SF-36 (PF, RF, BP, GH, VT, SF, RE, and MH) were lower in the SH group than in the non-SH group. Among the K-SF-36 subscale scores, PF, RP, BP, and SF scores were lower in women than in men, indicating that women were more susceptible to a reduction in HRQoL and especially physical HRQoL. Despite gender differences in some of the HRQoL subscales, there was no interaction between gender and SH observed in HRQoL scores.

**Table 5:** Differences in the HRQoL levels between the SH and non-SH groups, and between genders (participants, n = 237).

Scale		Subscale	Group	Gender		Source	*F*	*P* value
				Male	Female			
GHQ-30			SH	11.96 ± 7.03	14.63 ± 7.65	Gender	3.268	0.072
						SH/non-SH	53.099	**< 0.001**
			non-SH	6.73 ± 4.93	7.19 ± 4.90	Gender*(SH/non-SH)	1.610	0.206
SF-36	Mental component	MH	SH	56.25 ± 18.07	50.33 ± 19.20	Gender	1.136	0.288
						SH/non-SH	42.195	**< 0.001**
			non-SH	68.40 ± 13.08	69.22 ± 15.56	Gender*(SH/non-SH)	1.991	0.160
		RE	SH	62.50 ± 46.43	47.77 ± 45.21	Gender	2.325	0.129
						SH/non-SH	30.801	**< 0.001**
			non-SH	85.53 ± 30.52	83.98 ± 29.42	Gender*(SH/non-SH)	1.522	0.218
		SF	SH	69.79 ± 22.69	60.00 ± 23.53	Gender	6.715	**0.010**
						SH/non-SH	22.218	**< 0.001**
			non-SH	81.84 ± 17.26	76.13 ± 18.60	Gender*(SH/non-SH)	0.468	0.495
		VT	SH	43.22 ± 15.84	36.87 ± 17.93	Gender	1.457	0.229
						SH/non-SH	25.238	**< 0.001**
			non-SH	52.83 ± 16.23	53.00 ± 16.20	Gender*(SH/non-SH)	1.624	0.204
	Physical component	GH	SH	55.21 ± 20.24	47.67 ± 18.74	Gender	1.992	0.159
						SH/non-SH	12.355	**0.001**
			non-SH	61.32 ± 17.53	61.04 ± 16.86	Gender*(SH/non-SH)	1.715	0.192
		BP	SH	70.83 ± 20.07	62.08 ± 19.27	Gender	4.477	**0.035**
						SH/non-SH	20.934	**< 0.001**
			non-SH	81.36 ± 17.78	77.92 ± 18.57	Gender*(SH/non-SH)	0.847	0.358
		PF	SH	87.29 ± 17.00	84.33 ± 13.37	Gender	5.484	**0.020**
						SH/non-SH	18.257	**< 0.001**
			non-SH	95.61±9.17	90.58±9.69	Gender*(SH/non-SH)	0.369	0.544
		RP	SH	65.63±39.57	46.67±38.13	Gender	4.804	**0.029**
						SH/non-SH	38.831	**<0.001**
			non-SH	85.85±26.50	84.42±26.91	Gender*(SH/non-SH)	3.548	0.061

HRQoL = Health-related quality of life, SH = Symptomatic hyperventilation, HRQoL = Health-related quality of life, KNQ = Korean version of the Nijmegen Questionnaire, K-GHQ-30 = Korean version of the General Health Questionnaire-30, K-SF-36 = Korean version of the Short Form-36, PF = Physical functioning, RP = Role-physical, BP = Bodily pain, GH = General health, VT = Vitality, SF = Social functioning, RE = Role-emotional, MH = Mental health. Bold values indicate statistically significant results (p < 0.05).

## Discussion

In this study, we developed a cross-cultural translation of the NQ in Korean, with translation, back-translation, and face validity testing [17]. The “feelings of anxiety” term, which was removed from the construct validity test of the original NQ [4], had a high factor loading value (0.730) in this study, indicating that it should be included in the KNQ. Among the four KNQ factors, the “neuropsychological” and “respiratory” factors corresponded to the “shortness of breath” factor in the original NQ, and the “neuromuscular” and “neurogastrointestinal” factors corresponded to the “peripheral tetany” and “central tetany” factors of the original NQ [4]. Therefore, it appears that the factorial structure of the original NQ was largely replicated in the KNQ.

Using a cut-off score of 23 points, 22.8% of the study participants were classified in the SH group, showing that a sizable proportion of this population experienced symptoms associated with hyperventilation. This prevalence was slightly lower than the 27% reported in a general female population [11], and was similar to the 21% reported in a children and adolescent population [12]. Considering the previous and present study results, it appears that the prevalence of SH is around 25% in the general population, including children, adolescents, and a young adult (college) population. It is interesting that these prevalences are higher than the 8% reported in a group of people with prior asthma disease [10]. One possibility is that the study by Thomas et al. included only ex-patients who had previously been treated intensively for other medical problems, and hyperventilation-related symptoms may have been alleviated, together with main problems. On the other hand, the young population in this study may have been less likely to seek treatment for hyperventilation-related symptoms if their daily lives were not substantially affected.

Two-way ANOVA results showed that females were more susceptible to a reduction in physical HRQoL than males and that the SH group experienced lower HRQoL than the non-SH group. However, there was no interaction found between SH and gender. This suggests that similar to SH prevalence, reductions in HRQoL from SH may occur equally in both genders, despite female susceptibility to reduced HRQoL. It has been suggested that the NQ screening test should be included when treating nasal congestion, because SH might be the main etiological factor of this problem [22]. Furthermore, they suggested that NQ screening might spare unnecessary procedures and prevent economic waste associated with the medical problem [22]. Like Hanna’s suggestion, our study results support the view that KNQ screening may reduce unnecessary procedures and economic waste in young people with low HRQoL levels associated with SH. Moreover, if conducted in a large population, the benefit of screening could be substantial. To improve HRQoL associated with SH, hyperventilation-focused treatment strategies, including respiratory, biomechanical, and psychological treatment methods, may be considered for clinical use [16].

In this study, we cross-culturally translated the NQ into Korean and showed that the resulting KNQ was reliable and valid. We also found a SH prevalence of 22.78% in a young college population and lower mental and physical HRQoL levels in the SH group than in the non-SH group. However, our study had some limitations. First, the subjects were college students, so there is limited generalizability. Second, the KNQ focuses on symptoms, and it is challengeable to examine the association between HRQoL and clinical severity of hyperventilation, on the basis of arterial CO_2_ level. Further studies are needed to address these concerns.

## Supporting information

S1 File Dataset of the study. KNQ = Korean version of the Nijmegen Questionnaire, K-GHQ-30 = Korean version of the General Health Questionnaire-30, K-SF-36 = Korean version of the Short Form-36.
(XLSX)

S2 File Korean version of the nijmegen questionnaire. (PDF)

S3 File Korean version of the general health questionnaire-30. (PDF)

S4 File Korean version of the short form-36. (PDF)
