# Supplementary material for: Association of dysfunctional breathing with health-related quality of life: A cross-sectional study in a young population
Source: PLoS One. 2018 Oct 11;13(10):e0205634. doi: 10.1371/journal.pone.0205634 (PMC6181383; doi:10.1371/journal.pone.0205634)
Supplement: S2 File — (PDF) [file pone.0205634.s002.pdf]

# 한국어판 네이메헨 설문지

(Korean Version of the Nijmegen Questionnaire)

## [기본사항]

| | | | |
|---|---|---|---|
| 성별 | 남 / 녀 | 나이 | 세 |
| 작성일시 | 년 월 일 | | |

## [작성방법]

1. 본 설문지는 개인의 증상을 체크하여 호흡실조를 평가하기 위한 설문지입니다.
2. 설문지는 총 16문항입니다.
3. 정답이 있는 문항이 아니기 때문에 자연스러운 마음가짐으로 응답해주시면 됩니다.
4. 아래의 0점~5점의 설명 중에서 본인의 증상에 제일 가깝다고 생각되는 칸에 √표시를 하면 됩니다.
5. 시간제한은 없으나 어느 한 문항을 너무 오래 생각하지 않는 것이 좋습니다.

| 아래 문항들은 평소(오늘을 포함하여 최근 1주일 이내) 자신이 느끼는 몸의 상태에 대한 질문입니다.<br>해당항목에 체크(√)를 해주십시오. | | | | |
|---|---|---|---|---|
| 0점 | 1점 | 2점 | 3점 | 4점 |
| 전혀 나타나지 않는다. | 아주 가끔 나타난다. | 가끔 나타난다. | 자주 나타난다. | 매우 자주 나타난다. |

| 설문항목 | 0 | 1 | 2 | 3 | 4 |
|---|---|---|---|---|---|
| 1. 가슴부위 통증 | | | | | |
| 2. 긴장된 느낌 | | | | | |
| 3. 시야가 흐릿함 | | | | | |
| 4. 현기증 (어지럼증) | | | | | |
| 5. 혼란스러워 이해나 판단이 어려운 느낌 | | | | | |
| 6. 숨(호흡)이 점점 더 빨라지거나 깊어짐 | | | | | |
| 7. 숨(호흡)이 짧음 | | | | | |
| 8. 가슴이 조이는 느낌 | | | | | |
| 9. 윗배 더부룩함 (팽만감) | | | | | |
| 10. 손가락이 따끔거리는 느낌 | | | | | |
| 11. 숨을 깊게 못 쉼 | | | | | |
| 12. 손가락이나 팔이 뻣뻣함 | | | | | |
| 13. 입 주위가 조이는 (당기는) 느낌 | | | | | |
| 14. 손이나 발이 차가움 | | | | | |
| 15. 심장이 두근거림 | | | | | |
| 16. 불안한 느낌 | | | | | |
