# Supplementary material for: Association of dysfunctional breathing with health-related quality of life: A cross-sectional study in a young population
Source: PLoS One. 2018 Oct 11;13(10):e0205634. doi: 10.1371/journal.pone.0205634 (PMC6181383; doi:10.1371/journal.pone.0205634)
Supplement: S3 File — (PDF) [file pone.0205634.s003.pdf]

The Korean General Health Questionnaire-30

□ 성별: 남　여　　　　　　　　　　　　□ 나이:

[작성 방법]

다음은 일반적인 정신건강상태 측정을 위한 설문지입니다.

당신이 일상 생활에서 주로 느낀 바에 관하여 가장 가깝다고 생각되는 응답항목을 골라서 체크(√)해 주시기 바랍니다.

No	설문 항목	① 매우 그렇다	② 그렇다	③ 아니다	④ 매우 아니다
1	하고 있는 일에 잘 집중할 수 있었습니까?				
2	걱정 때문에 잠을 잘 못 잔 적이 많았습니까?				
3	자신이 여러 면에서 쓸모 있는 역할을 잘 하고 있다고 느꼈습니까?				
4	매사에 올바른 결정을 잘 내릴 수 있었습니까?				
5	계속해서 긴장감을 느낀 적이 자주 있었습니까?				
6	어려움을 극복할 수 없다고 느낀 적이 있습니까?				
7	일상적인 활동을 즐겁게 할 수 있었습니까?				
8	문제가 생기면 그것을 피하지 않고 맞서서 해결하려고 하였습니까?				
9	우울감과 불행감을 느낀 적이 많습니까?				
10	자신감이 없어졌다고 느꼈습니까?				
11	그 전과 같은 정도로 외출을 하였습니까?				
12	인생이 절망적이라고 느낀 적이 있습니까?				
13	신경이 쓰여서 힘들게 느껴진 일이 많았습니까?				
14	밤에 잠을 잘 못 이루거나 많이 설치곤 합니까?				
15	어떤 일을 할 때 대부분의 다른 사람들이 하는 만큼 그 일을 잘 처리할 수 있었습니까?				

No	설문 항목	① 매우 그렇다	② 그렇다	③ 아니다	④ 매우 아니다
16	당신과 가까운 사람들에게 따뜻함과 애정을 느낄 수 있었습니까?				
17	자신의 미래를 회의적으로 느꼈습니까?				
18	부지런히 생활하며 일에 전념할 수 있었습니까?				
19	다른 사람들과 잘 지내는 것이 어렵지 않게 느껴졌습니까?				
20	전반적으로 자신이 일을 잘 하고 있다고 느꼈습니까?				
21	대체적으로 행복하다고 느꼈습니까?				
22	사람들과의 대화시간을 많이 가진 편입니까?				
23	인생이 항상 고생스러운 것으로 생각되었습니까?				
24	자신이 가치 없는 사람이라는 생각을 자주 하였습니까?				
25	자신의 일을 처리해 온 방법에 만족하십니까?				
26	모든 일이 한꺼번에 당신을 힘들게 한다고 느낀 적이 있습니까?				
27	언제나 초조감이나 긴장감을 느끼는 편이었습니까?				
28	때때로 신경이 쇠약해져서 아무 것도 할 수 없던 적이 있습니까?				
29	별 뚜렷한 이유도 없이 공포와 두려움을 느낀 적이 많습니까?				
30	인생이 살 가치가 없게 느껴진 적이 있습니까?				

- 경희대학교 한방병원 진단•생기능의학과 -
