# Supplementary material for: Association of dysfunctional breathing with health-related quality of life: A cross-sectional study in a young population
Source: PLoS One. 2018 Oct 11;13(10):e0205634. doi: 10.1371/journal.pone.0205634 (PMC6181383; doi:10.1371/journal.pone.0205634)
Supplement: S4 File — (PDF) [file pone.0205634.s004.pdf]

건강관련 삶의 질에 관한 설문조사 (SF-36)

□ 성별: 남 여 □ 나이:

[작성 방법]

다음은 건강과 관련된 삶의 질 측정을 위한 설문지입니다.

아래의 모든 문항에 대해 당신이 최근 한 달 동안 주로 느낀 바에 관하여 가장 가깝다고 생각되는 응답항목을 골라서 □에 √표시를 해 주시기 바랍니다.

1) 평소 당신의 건강 상태는 어떻다고 생각하십니까?

아주 매우 좋다	매우 좋다	좋다	보통이다	나쁘다
□	□	□	□	□

2) 작년과 비교하여, 현재 당신의 건강은 어떤 상태라고 생각하십니까?

작년보다 아주 건강하다	작년보다 건강하다	작년과 비슷하다	작년보다 건강하지 못하다	작년보다 아주 건강하지 못하다
□	□	□	□	□

* 다음은 평상시 당신의 활동에 관한 항목들입니다. 이러한 활동을 할 때 지장이 있으십니까? 있다면 어느 정도입니까?

	지장이 많다	지장이 약간 있다	지장이 전혀 없다
3) 달리기, 무거운 물건 들어올리기, 격렬한 운동을 할 때	□	□	□
4) 탁자 옮기기, 집안 청소, 배드민턴 치기	□	□	□
5) 시장보기	□	□	□
6) 한 번에 두세 계단 오르기	□	□	□
7) 한 계단씩 오르기	□	□	□
8) 구부리기, 무릎 꿇기	□	□	□
9) 4~5리(1km 정도) 걷기	□	□	□
10) 마을 주변 산책, 동네 한바퀴 걷기	□	□	□
11) 집 주변 걷기	□	□	□
12) 혼자서 목욕이나 옷 입기	□	□	□

* 지난 한 달간 당신은 신체적인 어려움으로 일상생활이나 직장에서 다음과 같은 어려움이 있었습니까?

	모든 시간을 그랬다	대부분의 시간을 그랬다	약간의 시간을 그랬다	조금의 시간을 그랬다	전혀 없었다
13) 일하는 시간을 줄여야만 했다	☐	☐	☐	☐	☐
14) 원하는 만큼 일하지 못했다	☐	☐	☐	☐	☐
15) 어떤 일을 할 때 힘이 들 때가 있었다	☐	☐	☐	☐	☐
16) 대부분의 일을 할 때 어려움이 있었다	☐	☐	☐	☐	☐

* 지난 한 달간 감정적인 어려움으로 일상생활이나 직장에서 다음과 같은 어려움이 있었습니까?

	모든 시간을 그랬다	대부분의 시간을 그랬다	약간의 시간을 그랬다	조금의 시간을 그랬다	전혀 없었다
17) 일하는 시간을 줄여야만 했다	☐	☐	☐	☐	☐
18) 원하는 만큼 일을 하지 못했다	☐	☐	☐	☐	☐
19) 어떤 일을 할 때 힘이 들 때가 있었다	☐	☐	☐	☐	☐

20) 지난 한 달간 신체상의 어려움이나 감정상의 어려움으로 사회생활(가족간, 친구간, 이웃간)에 어려움이 있었다면 어느 정도입니까?

전혀 없었다	약간 있었다	보통 있었다	꽤 많이 있었다	매우 많이 있었다
☐	☐	☐	☐	☐

21) 지난 한 달간 당신의 신체적인 아픔은 어느 정도 였습니까?

전혀 없었다	아주 약하게 있었다	약하게 있었다	보통 있었다	심하게 있었다	매우 심하게 있었다
☐	☐	☐	☐	☐	

22) 신체적 아픔으로 인해 정상적인 생활에 지장이 있습니까?

전혀 없었다	약간 있었다	보통 있었다	꽤 많이 있었다	매우 많이 있었다
☐	☐	☐	☐	☐

* 다음의 질문들은 지난 한 달간 여러분이 어떻게 느꼈고, 어떤 일이 있었는지에 관한 것입니다. 각각의 문항에 대하여 근접한 대답을 하나씩 답하시오.

	모든 시간을 그랬다	대부분의 시간을 그랬다	약간의 시간을 그랬다	조금의 시간을 그랬다	전혀 없었다
23) 나는 의욕이 넘쳤다	☐	☐	☐	☐	☐
24) 나는 신경질적 이었다	☐	☐	☐	☐	☐
25) 나는 의기소침 했었다	☐	☐	☐	☐	☐
26) 나는 안정되고 평온했다	☐	☐	☐	☐	☐
27) 나는 힘이 넘쳤다	☐	☐	☐	☐	☐
28) 나는 우울하고 상심했었다	☐	☐	☐	☐	☐
29) 나는 지쳤었다	☐	☐	☐	☐	☐
30) 나는 행복 했었다	☐	☐	☐	☐	☐
31) 나는 피곤했었다	☐	☐	☐	☐	☐

32) <u>지난 한 달간 몸이 불편하다든지 아니면 기분이 내키지 않아서</u> 친척이나 친구 등을 방문하는 것과 같은 일을 하는데 어려움이 있었다.

모든 시간을 그랬다	대부분의 시간을 그랬다	약간의 시간을 그랬다	조금의 시간을 그랬다	전혀 없었다
☐	☐	☐	☐	☐

* 다음의 질문에 답해 주십시오.

	매우 사실이다	사실이다	모르겠다	거짓이다	매우 거짓이다
33) 나는 다른 사람들보다 더 쉽게 병에 걸리는 것 같다	☐	☐	☐	☐	☐
34) 나는 누구보다도 건강하다	☐	☐	☐	☐	☐
35) 나의 건강은 점점 나빠질 것이다	☐	☐	☐	☐	☐
36) 나의 건강상태는 매우 좋다	☐	☐	☐	☐	☐

- 경희대학교 한방병원 진단•생기능의학과 -
